# Advancing clinical decision support using lessons from outside of healthcare: an interdisciplinary systematic review

**DOI:** 10.1186/1472-6947-12-90

**Published:** 2012-08-17

**Authors:** Helen W Wu, Paul K Davis, Douglas S Bell

**Affiliations:** 1 RAND Corporation and Pardee RAND Graduate School, 1776 Main St, Santa Monica, CA, USA; 2 David Geffen School of Medicine, University of California, Los Angeles, CA, USA

## Abstract

**Background:**

Greater use of computerized decision support (DS) systems could address continuing safety and quality problems in healthcare, but the healthcare field has struggled to implement DS technology. This study surveys DS experience across multiple non-healthcare disciplines for new insights that are generalizable to healthcare provider decisions. In particular, it sought design principles and lessons learned from the other disciplines that could inform efforts to accelerate the adoption of clinical decision support (CDS).

**Methods:**

Our systematic review drew broadly from non-healthcare databases in the basic sciences, social sciences, humanities, engineering, business, and defense: PsychINFO, BusinessSource Premier, Social Sciences Abstracts, Web of Science, and Defense Technical Information Center. Because our interest was in DS that could apply to clinical decisions, we selected articles that (1) provided a review, overview, discussion of lessons learned, or an evaluation of design or implementation aspects of DS within a non-healthcare discipline and (2) involved an element of human judgment at the individual level, as opposed to decisions that can be fully automated or that are made at the organizational level.

**Results:**

Clinical decisions share some similarities with decisions made by military commanders, business managers, and other leaders: they involve assessing new situations and choosing courses of action with major consequences, under time pressure, and with incomplete information. We identified seven high-level DS system design features from the non-healthcare literature that could be applied to CDS: providing broad, system-level perspectives; customizing interfaces to specific users and roles; making the DS reasoning transparent; presenting data effectively; generating multiple scenarios covering disparate outcomes (*e.g.,* effective; effective with side effects; ineffective); allowing for contingent adaptations; and facilitating collaboration. The article provides examples of each feature. The DS literature also emphasizes the importance of organizational culture and training in implementation success. The literature contrasts “rational-analytic” *vs.* “naturalistic-intuitive” decision-making styles, but the best approach is often a balanced approach that combines both styles. It is also important for DS systems to enable exploration of multiple assumptions, and incorporation of new information in response to changing circumstances.

**Conclusions:**

Complex, high-level decision-making has common features across disciplines as seemingly disparate as defense, business, and healthcare. National efforts to advance the health information technology agenda through broader CDS adoption could benefit by applying the DS principles identified in this review.

## Background

Healthcare lags behind many other disciplines in the diffusion of technology. In the area of computer-based decision support (DS), other disciplines have been using DS systems since the 1970s [1]. In the fields of defense, energy, environment, finance, business strategy, and public policy [2] tools such as knowledge management, expert systems, exploratory analysis, and data mining are ubiquitous. Although the need for DS to support clinical decision-making is considerable, the spread of health information technology (HIT) with Clinical Decision Support (CDS) in U.S. healthcare has been slow [3].

While both clinical and non-clinical DS systems have faced implementation challenges, lessons learned from other disciplines, particularly those in which DS use is more widespread, could inform efforts to advance the adoption of CDS. Interdisciplinary approaches when used in health services research, can be useful in finding solutions that can generalize across problems of a similar fundamental nature, identifying the full complexity of problems, and finding new insights [4]. Patient-safety initiatives have benefitted by applying strategies from commercial aviation to reduce medical errors [5]; likewise, HIT efforts could benefit from an interdisciplinary examination of DS applications.

Current CDS systems vary widely in their structure and function, ranging from medication dosing support to triage tools to workflow planning [6], and this variation is present in non-healthcare disciplines as well. We do not make comparisons between specific types of clinical and non-clinical DS systems (*e.g.*, computerized-physician-order entry *vs.* an analogous non-clinical tool), but instead aim to synthesize general lessons learned from the body of literature on design features of DS tools in relevant, non-healthcare applications.

Previous research has documented common features of successful CDS. Kawamoto et al. described 15 features that were repeatedly found in the literature; of these, 4 were statistically associated with success (integration with workflow, giving recommendations rather than assessments, giving DS at the time and place of decision-making, and giving computer-based DS) [7]. Mollon et al. identified 28 technical, user interaction, logic-based, and developmental/administrative environment features [8]. Bates et al. used experiences in CDS to recommend ten principles for effective CDS design [9]. The existing research focuses on design features and policy recommendations to encourage adoption [10].

Early CDS efforts arose from the same Bayesian reasoning principles that gave rise to early DS tools in non-healthcare disciplines [11]. However, these early CDS tools did not find their way routine clinical practice. The degree to which subsequent CDS efforts were informed by non-healthcare experience is unknown, but we are not aware of explicit discussions of how and why non-healthcare applications might be transferrable. This review contributes to the literature by summarizing broad lessons learned, based on a fresh, and perhaps novel, interdisciplinary look outside of the clinical realm. In non-healthcare research, Burstein and Holsapple compiled examples of DS systems and applications [12,13], but not as a comprehensive review. We found no studies that provided a review of how other disciplines’ experience with DS could inform CDS.

### Objective

Our goal was to survey the literature on DS system design and implementation in non-healthcare disciplines, in order to contribute to our understanding of how to accelerate the adoption of CDS. We summarize key successes, best practices, and lessons learned from non-healthcare decision-making processes, and we identify tools that may inform the design of CDS. The primary focus is on DS systems, but as systems are only as good as the foundations they are built upon, this paper also briefly describes literature on decision-making principles.

Healthcare CDS has been defined as: *"Health information technology functionality that builds upon the foundation of an electronic health record (EHR) to provide persons involved in care processes with general and person-specific information, intelligently filtered and organized, at appropriate times, to enhance health and health care"*[14]*.* Our intent was, in reviewing non-healthcare DS, to take a broad view that would include applications based on artificial intelligence, knowledge management, neural networks, collaborative decision support, expert systems, and other methods.

## Methods

This study was an interdisciplinary systematic review of literature pertaining to DS systems in non-healthcare disciplines. Interdisciplinary research can provide valuable new insights, but synthesizing articles across disciplines with highly varied standards, formats, terminology, and methods required an adapted approach. The study methodology applied the basic framework used for systematic reviews in healthcare [15]. We: 1) defined a scope of decisions and DS types that might be generalized to healthcare, 2) identified appropriate databases and performed searches within a defined timeframe for a fixed set of search terms, 3) selected abstracts for full-text review based on multiple reviewer agreement, 4) developed selection criterion to exclude lower-quality articles, and 5) reviewed the remaining articles for common themes of interest, abstracting qualitative data. Our generalization of healthcare systematic review methods approach was similar to that used by Evans and Benefield in examining educational research using a healthcare systematic review process [16].

With the aid of a reference librarian who identified relevant sources, in August 2010, we searched a number of databases for peer-reviewed and grey literature work published in 2005–2010 broadly representing basic sciences, social sciences, humanities, engineering, and business disciplines. The databases were PsychINFO, BusinessSource Premier, Social Sciences Abstracts, and Web of Science. Search terms were narrowed using an iterative process, based on the number of results returned and a review of a sample of each set of results for relevance. The final search terms were: decision support, decision system, and expert system; broader search parameters yielded too many irrelevant results. Articles with terms related to healthcare topics in the title or abstract were excluded. We also used a comprehensive database for unclassified defense work, the Defense Technical Information Center (DTIC). Using DTIC required a modified search strategy: we extended the timeframe to be from 2000–2010 in order to capture more reports, added “commander” to the search terms so as to exclude documents about low-level tactical decisions not generalizable to physicians, and relaxed constraints on publication type because relatively little of the relevant high-quality information in defense-sponsored work is disseminated in journals.

The literature on DS is enormous, and a large proportion of articles were descriptions of a single system from the subjective, and possibly biased, viewpoint of developers or users. Given the wide variability in article quality and format across multiple non-healthcare disciplines, we applied criteria that selected for more objective review-style work and for decision support relating to the high or moderately high-level decisions akin to those made by physicians. The *first selection criterion* was that the work was a review, overview, discussion of lessons learned, or evaluative piece across a discipline, or for a design or implementation aspect of DS. This was an indirect strategy for assessing DS system success or quality, as those included in reviews were felt to represent at least some level of success. The *second criterion* required the work to describe decisions with an element of individual human judgment, to ensure comparability to CDS. This excluded systems that fully automated decisions, such as models and systems related to optimization, industry / manufacturing, and systems engineering. We also excluded systems for which the object of decision-making was the organization, such as those dealing with organizational finance, long-term strategy, forecasting, or monitoring, as this also does not generalize well to clinical decisions for patient care. We abstracted findings related to one of three themes:

1) Characteristics of decisions being supported *–* general features of the decisions that the systems in question were designed to support, with a focus on areas of similarity between healthcare and non-healthcare settings.

2) Principles used in decision-making – theoretical approaches about how to structure and make decisions, which in turn could be used to conceptualize DS tools.

3) Design features of DS tools – lessons learned, successful examples, and recommendations on how to improve the design and implementation of non-clinical DS aids, tools, and systems.

The initial search of five databases generated 890 unique results covering a wide range of disciplines. Of this set, one study author reviewed the titles and abstracts to identify a preliminary set that met the selection criteria, identifying 74 for further review. These 74 articles represented many disciplines, including environment, agriculture, natural resource management, urban planning, defense, law, and business disciplines. The other two study authors reviewed the preliminary set and identified 38 articles that met both selection criteria for full-text review. Where there was disagreement between authors, we erred on the side of inclusion. During the full-text review, 11 articles were excluded since they did not meet the two inclusion criteria or did not have any information related to these three themes of interest, leaving a final sample size of 27 references represented in this review. Of these 27 references, 9 related to defense topics [17-25], 2 related to business [26,27], 1 related to law [28], and 15 related to decision science generally [29-43]. The initial set of articles included a broad range of disciplines, but the articles that passed the selection criteria for study quality and relevance, and for included content related to one the three common themes, were from a smaller set of disciplines. We also included a few select works that we knew were highly relevant from our expertise in decision making theory, but that did not appear in the literature search due to limitations of the search strategy [14,44-48]. The additional references sharpen key concepts and principles that did, however, arise in the initial search.

## Results

### Decision characteristics

Although the content of decisions varies across disciplines, the nature of the decisions and the DS tools used to inform them may have similarities. Table 1 compares similar characteristics of clinical and non-clinical decision parameters, with the purpose of setting the theoretical framework for subsequent DS tool development. Clinical and military decision making may appear to be polar opposites on the surface, but the examples in Table 1 show how high-level decision makers face many of the same types of challenges. Namely, they must make decisions that: 1) are urgent and require a quick initial response, 2) require rapid adaptation to changing circumstances and new information, 3) have life-or-death consequences, 4) have uncertain responses from opponents/adversaries due to incomplete, imperfect information and unpredictable behavior, and 5) require an understanding and ability to synthesize the interaction of various risks. DS tools serve to operationalize decision making approaches, so it is important to view decision characteristics as the framework for subsequent nuts and bolts comparisons of specific tools.

**Table 1:** Analogies between clinical and defense decisions

**Decision need**	**Clinical example**	**Defense example**
Urgency, need for rapid initial response	*Emergency department triage*. Triage requires near-instantaneous decisions, in order to avoid delaying critical treatment for high-priority patients [49].	*Command centers*. The air force command center, which controls all available aircraft, must decide which requests for air support from ground forces in trouble get priority, with timeliness sometimes being essential.
Adaptation to changing circumstances, new information	*Ventilator management*. As patient respiratory function changes, ventilators must adjust accordingly [50].	*Improvised explosive device (IED) disarmament*. The tactics and technology of IEDs changes over a period of weeks, so the strategy to disarm them must do so also. DS includes real-time surveillance and computer models that help anticipate adversary actions.
High consequence, life or death implications	Many, for example cancer chemotherapy order sets, dose checking; radiation therapy planning.	*Mission choice*. Send troops on a dangerous mission from which they may not return. The decision to send a SEAL team in to get bin Laden risked lives of the team and international crisis, but was in pursuit of a compelling objective.
Uncertain possibilities due to incomplete, imperfect information	*Diagnostic expert systems*. Diagnosis relies upon accurate assessment of signs and symptoms, but these do not always provide reliable information, such as when the patient is unable to communicable effectively.	*War planning*. Enemy behavior cannot be predicted with full precision, information on the enemy is incomplete [20] and imperfect, with deep uncertainty [18]. War planners must anticipate and be ready to deal with many adversary tactics. In the 2003 war with Iraq, U.S. forces prepared for chemical-weapon attacks, mass movement of refugees, burning of oil facilities, etc.
Balancing disparate types of risks and benefits	*Treatment selection*. The effectiveness of adjuvant chemotherapy, which has its risks and side effects, depends on patient factors and tumor stage [51].	*Attacks near population centers*. Air force attacks must evaluate how weapons used affect target accuracy, the risk of civilian casualties, and effectiveness. This balances risks of collateral damage, international incident, and effectiveness. DS includes accurate computer maps, weapon-effects models, and rigid doctrine and discipline.

Cognitive biases are also similar across clinical and non-clinical decision-makers. Individuals ranging from military commanders to manufacturing floor managers to trial juries may be subject to cognitive distortions that include availability, representativeness, anchoring and adjustment, and confirmation biases [17,28,33,37]. Humans’ probabilistic reasoning ability is poor, and both physicians and patients may inaccurately interpret single probabilities, conditional probabilities, and relative risks when making decisions [52]. An objective, accurate understanding of the complete situation – often termed “situational awareness” or “the common operating picture” in defense – can mitigate these biases, which would otherwise lead the decision-maker down the wrong path [17,22]. Likewise, new clinical diagnoses must be made in the context of the patient’s overall medical history as opposed to a purely problem-focused evaluation; otherwise, the problem may be misidentified.

### Principles of decision-making

Decision support is only as good as its underlying foundation, and an understanding of how to frame decision-making, regardless of how or whether DS tools are used, is a useful starting foundation. Principles of decision-making identified in the review address: 1) the distinction between rational-analytic *vs.* naturalistic-intuitive decision-making styles, 2) the utility of a flexible, adaptive, robust, approach that considers multiple criteria and possibilities (often reflected as alternative scenarios), 3) and the notion of appropriate levels of trust in recommendations.

Two general approaches to decision-making have taken turns in the spotlight over time [53], described by Davis, Kulick and Egner [44] and Davis and Kahan [18] as the *rational-analytic* and *naturalistic-intuitive* styles. The former draws upon data, models, and breadth-first evaluation of a set of options to make decisions, whereas the latter exploits experience and judgment. Rational-analytic decision-making drove much of the initial development of DS systems [22]. It is useful in synthesizing large amounts of information and mitigating some cognitive and intellectual biases, but it is limited by narrowly defined computer-based rules and models, which cannot adapt to contextual, big-picture considerations [17,18,29]. Naturalistic-intuitive decision-making, on the other hand, considers a human element to be critical even in situations highly amenable to automation, as people can identify novel patterns, exploit synergies, and be more creative – i.e., finding solutions that would not have been found through procedural reasoning within a narrow methodology. However, it may lead to preventable error and is prone to cognitive and other biases. Even in an industrial manufacturing application, which is highly amenable to automation, Metatioxis noted that “the production manager is the expert who knows the whole production environment and its special features and who can handle changing and unexpected events, multiple objectives and other similar aspects where human flexibility is necessary” [35].

**Figure 1:**
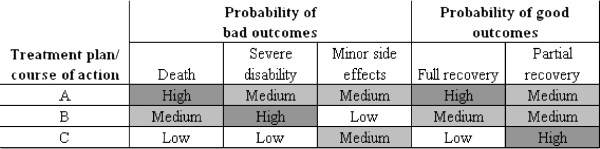
Example of rational-analytic and naturalistic decision styles combined.

A well-rounded approach draws upon the strengths of each decision-making style, while minimizing the respective weaknesses [18]. Figure 1 provides an example of one way of framing decisions, which could be used in a DS system, that hypothetically uses rational-analytic methods to generate a *set* of scenarios that could in turn, be selected based on naturalistic-intuitive reasoning. Multi-scenario analysis such as this uses rational analysis not to optimize and provide a single answer, but to provide the decision-maker with a broad view of multiple hypotheses [22], relevant systems [20], or “branches and sequels” of possibilities [19]. As stated by Davis and Kahan, “high-level decision makers are commonly afflicted with deep uncertainties that materially affect the choice of a course of action … the solution is to adopt a course of action that is as flexible, adaptive, and robust as possible” [18]. Decisions that depend upon the validity of a single set of assumptions and criteria are not robust to uncertainties [18] and allow little room for judgment [33]. The key in DS system design is to provide enough information to give decision-makers a comprehensive view that mitigates the “fog of war” but not to overload them with so much information that it creates a “glare of war” – i.e., information overload [22,45,46].

Additionally, options generated by a DS system must be viewed with the *appropriate level of trust* – *e.g.*, about whether the system performs as expected, or whether it has the right content and structure to provide accurate and appropriate recommendations for the situation. Some decision-makers may place inappropriately low levels of trust in DS systems, due to fear of technology and automation, concern about the reliability of the overall system or its recommendations, or discomfort with black box methodologies [23,24,26]. Other users may be overly confident about the system’s ability, in which case the value of their intuitive expertise is diminished.

### Decision support system design features

In problem domains where DS is expected to be valuable, developers in non-healthcare fields have devised successful strategies for handling many problematic characteristics of decisions, while also paying attention to the principles of decision-making just discussed. These may be described in the literature as successes, lessons learned, or recommendations for improvement. They are organized in Table 2 as specific features of the design and implementation of DS tools, aids, and systems – ranging from non-technical decision aids to fully networked systems. The design features that emerged from the literature as keys to successful DS are elaborated upon in Table 2 and were grouped into six categories:

1) DS that provides broad, system-level, big picture perspectives helps to mitigate tunnel vision and support informed decision-making;

2) DS customized to users, settings, and purposes improves performance, whereas generic systems tend to perform poorly in specific problem domains;

3) DS protocols must be transparent in order to gain the appropriate level of user confidence;

4) DS must organize and present data effectively in order to fully mitigate the cognitive barriers to processing large volumes of information;

5) DS should allow users to generate/view multiple scenarios and options simultaneously in order to reap the benefits of both rational-analytic and naturalistic-intuitive decision-making; and

6) Collaborative, group-based DS should be designed to draw upon multi-disciplinary expertise

**Table 2:** Design features of non-clinical decision support applications

**Design feature and problems addressed**	**Key lessons from the literature**
***Feature: *Broad, system-level views of the big picture**	● Provide a broad overview, so that the decision-maker can see the entire environment, what is known, and what is not [22]. Develop a comprehensive view of operations and interconnected systems by identifying key nodes within each system (persons, places, or things), establishing relationships, emphasizing baseline data for the current situation and how it relates more generally to the known solutions, and categorizing information objectively [17]. Provide multiple levels of detail (i.e., the broad view with zooms) [45].
*Problems addressed:* Tunnel vision, cognitive biases that prevent consideration of the full range of options (*e.g.*, representation heuristic, anchoring and adjustment)	
	● Filter out unnecessary clutter to increase the leader’s situational awareness, and allow him/her to focus on key tasks [22], but allow system drill-down with increasing granularity to educate decision-makers on the task [33].
*Parallels in CDS literature:* None	
	● Frame problems with all the relevant factors and friendly/opposing viewpoints, posing questions throughout the process that prompt users to search for the root of the problem and think about what is not known [17]. Continue the problem formulation process until an opposing view is considered [37,42].
***Feature: *Customized to address specified problems and user needs**	● Development should balance virtues of careful initial design and rapid prototyping [47]. Tools that are simplified and customized for niche uses may, in some instances, be developed rapidly and avoid unnecessary complexity. Expanding a niche system to other user groups then requires a significant jump, and should be done after the processes, data formats, and availability are evaluated [27].
*Problems addressed:* Generic systems with too much complexity, which are not user-friendly do not handle any single problem well	
		● Commercial, off-the-shelf systems may work, but they need to be adapted appropriately to the targeted users [25,32]. In some situations, fully customized systems are required [22]. They should be part of an integrated information system, follow standard software development processes (developing, testing, maintenance), and use standards-consistent hardware and software platforms for acceptability, reliability and maintenance [35].
*Parallels in CDS literature:* Addressed somewhat by Bates [9]		
	● Different situations may demand different tools. A defense operation involves many phases: planning, deployment, execution, recovery, and post-operation work – and different tools are needed at each phase [19].	
***Feature: *Involving users in system design**	● Partner with end users in problem discovery and design [26,27]. User participation in the development phase can improve the success of adoption, in terms of user satisfaction, intent to use systems, and actual use of systems [30].	
*Problems addressed*: Poor adoption of system, user trust, ease of use		
*Parallels in CDS literature:* Addressed in many studies; see Kawamoto [7]		
***Feature: *Transparency that documents the underlying methodologies and decision processes**	● Ensure that users can apply their own judgment and explore trade-offs by using interactive tools and visuals to show likely/unlikely possibilities, short- and long-term trends, etc. – give “better answers, not just *the* answer,” including supporting evidence and key drivers of outcomes. Show how trade-offs between competing objectives affect outcomes [27], and provide the right level of granularity to back up recommendations [23]. “Build insight, not black boxes” [27].	
*Problems addressed:* User acceptance and over- or under-trust of system recommendations, “satisficing” behavior, ethical biases		
	● Collect metadata – data that describes the nature of the data, such as user actions and date/time stamps. Build in system capabilities that show what actions are recommended, when they were taken, and what criteria were satisfied to justify those actions. This facilitates tracking how the decision was made, and can be used to improve decisions or provide liability protection [19,22].	
*Parallels in CDS literature:* Partial: Kawamoto addressed “justification of decision support via provision of reasoning and evidence” [7]		
	● Elicit the decision-making structure [39]. Provide information about the reliability of the decision aid, and about the reliability of human judgment, to encourage appropriate use of systems – *e.g.*, avoiding blind adherence (overuse) and distrust (underuse) [24]. Restate issues and build flow diagrams that challenge the user to consider how each piece of evidence supports their decision [37].	
***Feature: *Effective organization and presentation of data**	● Use presentation methods such as summary dashboards, graphics and visuals, interactive simulations and models, storyboards, matrices, spreadsheets, qualitative data fields, and customized interfaces [25,26,33,37,39,42]. The most effective presentation format depends on the situation, and research does not consistently support which is right in which situation [39].	
*Problems addressed:* Cognitive limits on processing large volumes of data, meaningful application of naturalistic-intuitive decision-making within rational-analytic DS systems		
	● Display patterns that are better recognized by humans than computers in showing a trend, and avoid asking users for extra information from unformatted text [22].	
	● Provide well-conceived default formats and easy restoration, but allow users to control and customize displays using scatter diagrams, bar charts, dashboards, statistical analyses, reports, etc. [32]. Organize data using filtering and retrieval functions that allow users to change the aggregation level from highly detailed to overall summaries, but add in alerts in case users filter out important information [22,32] – i.e., allow users to “pull” extra information as desired.	
*Parallels in CDS literature:* Partial: Topic of “relevant data display” in Wright [6]		
	● “Push” key information and updates to users – deliver prompts when critical new pieces of information arrive, tailored to the action requirements of specific users, and develop pre-programmed sets of plans that can be applied in response to new information [21]. Good DS design will push out only key information that facilitates the task, not overwhelm the user with too much information.	
	● Use consistent standards and terminology so that words, situations, and actions are clear, and to increase user friendliness [21].	
***Feature: *Multi-scenario, multi-option generation**	● Use multi-scenario generation, portfolio analysis, foresight methods, and branch-and-sequel methods to educate the decision-maker on the implications of uncertainty and ways to hedge, including with planned adaptation [18]. Use rational-analytic structures to assure presence of alternative choices and (possibly to apply probabilities and weights), but avoid making a single recommendation about the final choice – instead, show how changes in variables or criteria affect assessments [18,39].	
*Problems addressed: *Co-existing presence of rational-analytic and naturalistic-intuitive decision-making, unreliable nature of optimization-based models		
	● Allow the user to explore various outcomes by generating a distribution of all plausible outcomes, accounting for both desired and undesired effects [20]. Simplify by grouping assumptions (including those about values and disagreements), so that users can more readily see how choice depends on “perspective” [45].^1^	
*Parallels in CDS literature:* None		
	● Work backward from the observed outcome. Map out the possible chains of events that could have led to the outcome [28]. Alternatively, identify the potential outcomes, then examine all the branches that could lead to those outcomes. Use a hierarchical / nested design to show DS rules that lead to different results [29]. Functionally, the point is to show what one would have to believe to get different results.	
***Feature: *Collaborative, group, and web-based systems**	● Leverage the Internet and email to support collaborative decisions that draw upon a range of expertise [36,40]. Share information on a central website, which includes access to analytic tools, databases, and websites for more information [21].	
*Problems addressed: *De-centralized information sources, team collaboration in decision-making, interoperability of systems, need for broad range and depth of expertise from individuals in disparate locations		
	● At the same time, recognize that expert opinion is often not nearly so reliable as often assumed. This is highly dependent upon details of knowledge elicitation [54].	
	● Assure a user-friendly design that requires little training and presents a clear picture of the important features of the situation [22]. With collaborative tools, facilitate rapid communication [1,21].	
*Parallels in CDS literature:* None		
	● For “wicked problems” with unclear solutions, use cognitive, dialogue, and process mapping methods to encourage brainstorming and organize a group’s ideas [34].	

^1^A “perspective” represents a way of looking at issues. In military analyses, perspectives might reflect different values or judgments about the real-world feasibility of competing military strategies (*e.g.*, counterinsurgency strategies based on U.S.-intensive efforts versus efforts to leverage indigenous forces of a supported government). In health care, by analogy, different perspectives might include maximizing length of life versus quality of life, or might reflect different assumptions about how well a patient could and would follow treatment recommendations. A number of assumptions would go naturally with each of these perspectives, reducing dimensionality of the uncertainty analysis. For publicly available decision-support software incorporating the perspectives methodology, see Davis PK and Dreyer P, RAND’s Portfolio Analysis Tool, Santa Monica, CA: RAND, 2009.

### Decision support system implementation

Good system design is an obvious prerequisite for effective implementation, as detailed in Table 2. Beyond design considerations, two overall themes emerged related to implementation: 1) the need for continual system improvements, and 2) effective user training that addresses individual and organizational issues during adoption. Although these are not new lessons for CDS research, their recurrence in non-clinical DS research should strengthen them as a priority in CDS. Also, we describe a few insights about how these issues are addressed in non-healthcare realms.

Continual improvement is important in non-clinical DS since it addresses the need for systems to be flexible, adaptive, and avoid perpetuating rules or data that are inaccurate or outdated. DS systems should be continually re-evaluated and fine-tuned, and implementation should not be viewed as a one-time task. Systems should never be viewed as final, since they must change frequently based on the nature of the problem, users, and environment. DS development should follow a three-part cycle – initiation, analysis, and delivery, which should be re-visited [33]. With respect to user training, DS implementation may fail because users are not properly trained on how to use the system, do not fully understand the system’s capabilities and efficient ways to take advantage of them, and do not understand their roles and responsibilities. The consequence is inappropriate or non-use of a tool. The user training process could avoid these problems by: informing users about the reliability of the decision aid to encourage appropriate levels of trust [24]; instructing users on the nature of change and teach them new behaviors in order to “unfreeze” them from old ways of thinking about DS [33]; encouraging continuous learning from the tool [35]; and providing realistic training simulations [26].

In addition to the content of the training, a strong theme was the importance of organizational support. Leadership should motivate decision-makers to use systems by: building an accepting, supportive, non-coercive environment, and encouraging the consistent, continued use of systems [43]. They should find a champion, an in-house expert who is also an end user and decision-maker, who will promote the system within the organization [27]. Finally, they should define roles and responsibilities of each user and decision-maker, clarify processes and expectations for using the tool within the current system, outline how the tool will measure performance, and identify incentives and rewards to use the tool or demonstrate a change in performance [27].

## Discussion

The non-healthcare literature on DS offers a wealth of information that can be used to advance CDS. Characteristics of many decisions are similar between high-level clinical and non-clinical decision-makers, with elements of complexity, high uncertainty, unpredictable adversaries, rapid change, and the potential for cognitive biases. The basic principles that guide decision-making across fields are also worth noting. Both the rational-analytic and naturalistic-intuitive decision-making styles offer benefits and drawbacks, and users should understand how much to trust rational-analytic systems, in addition to being aware of naturalistic-intuitive biases.

This review focused on successes and best practices related to decision-making in non-healthcare disciplines. The literature consistently supports the notion that optimal DS system design depends heavily on situational factors – *e.g.*, the state of the knowledge and sciences, as well as organizational, political, and cultural context. Tools must be developed that solve the right problem – “a knowledge base for bridge design is not useful if it's unclear a bridge is needed at all" [34]. Although the literature provides common lessons learned about how to design effective DS, there is no single “best” design, as this depends on the needs of users, nature of the problem, and system context. The key lessons summarized in Table 2, in particular, should not be viewed as a prescription for how CDS should be designed, but rather used to reinforce the use of similar approaches in CDS, and understand what else might work. Implementation of CDS systems is also crucial. It must consider how individual factors and work processes motivate proper use, and how to set the right organizational context to support uptake. Although those working in CDS implementation may already be well aware of this, the non-healthcare literature underscores the notion that no matter what discipline, good system design is necessary but not sufficient for success.

Complex decisions call for a combination of naturalistic-intuitive and rational-analytic approaches. CDS based on rational-analytic methods, such as artificial intelligence technologies in medicine, must still incorporate intuitive judgments to be useful – a balanced approach. Whether these analytic approaches are embedded into computerized DS systems or not, decision-makers can draw upon the benefits of both approaches by prioritizing strategies that are flexible, adaptive, and robust (i.e., flexible enough to accommodate changes of mission or objective, adaptive to circumstances, and robust to adverse shocks). Having a suitable strategy of this sort is of little help, however, unless the effort is made to gain information as treatment proceeds, and to review and modify strategy accordingly. Thus, another principle of decision support should be to define a program for monitoring, information collection, review, and adjustment. In a clinical setting, such a program might include: (1) a written plan (even if informal) with anticipatable branches and potential surprise events, either good or bad, noted, (2) follow-up procedures to check on patient outcomes after decision support is used, (3) monitoring the evolution of knowledge, via colleagues and other experts, that may prove relevant to the treatment program over time, (4) scheduled laboratory tests and examinations, (5) organized big picture reviews that encourage fresh “rethinking” of the problem and the re-direction of treatment approaches if small adjustments to the current ones are not working. Computerized aids could help with most of these, whether or not they are seen as “decision aids.”

### Limitations

Our interdisciplinary review process offered a novel approach to examining a problem within the healthcare discipline, but interdisciplinary research also poses many known challenges [4]. We targeted a high-priority and relevant subset of a diverse but inconsistent literature, a process that inevitably overlooked some experiences and publications. Although DS has been used in fields such as aviation, emergency management, nuclear energy, agriculture, and environmental planning [12,13,33], our quality and relevance-based selection criteria yielded applicable work from only the disciplines of defense, law, and business, with only 27 of 890 (3.0%) of the search results being abstracted. This article selection rate is similar to Eom and Kim’s DS system survey, which retained 210 of 5400 (3.9%) articles [33]. Although the number of articles and disciplines selected for full-text extraction was limited, the value of the result should probably be judged by whether the conclusions are helpful in considering clinical DS, rather than by whether some additional conclusions might have been found with an even more exhaustive search.

We did not evaluate the effectiveness of specific DS systems described in the literature, but used the inclusion of the study in a review-type article as an indicator of quality or importance. This approach was necessitated by the challenges of interdisciplinary-based systematic review. Descriptions of study quality, outcomes, and measures of DS success could not be reliably abstracted, as is typical in systematic reviews of healthcare. Also, the work represented in this review is not comprehensive or representative of the universe of DS applications, but rather a sample of recent, available work related to high-level, complex decision-making. For example, a number of studies on and tools for strategic planning address issues related to decision making but are not discussed at length due to the focus of this paper [45,48]. Our study also does not represent the heterogeneity in DS function and purpose, whether clinical or non-clinical, but aims to distill lessons up to a sufficiently high level to be valuable for all types of CDS systems. A more extensive review process that included non-review articles and developed a methodology to evaluate the quality of DS “success” models in other fields would be valuable in future CDS research.

## Conclusion

Much has been written recently about how to remove barriers to the adoption of CDS as part of a broader national HIT agenda. These efforts would benefit from taking the view that clinical decisions have similarities to high-level decisions in many non-healthcare fields, and that HIT design can benefit from the long history of DS development and implementation outside the healthcare realm. In summary, we found that CDS systems may be better designed to support complex healthcare decisions if they consider the following features: providing broad, system-level perspectives; customizing interfaces to users and purposes; making protocols transparent; organizing and presenting data effectively; providing multiple scenario generation ability; and facilitating collaboration where appropriate. Moreover, as systems are only as good as the principles upon which they are built, it is crucial for both CDS users and developers to consider how to apply both rational-analytic and naturalistic-intuitive approaches to complex healthcare decision-making.

## Competing interests

The authors declare that they have no competing interests.

## Authors’ contributions

All the authors read and approved of the final manuscript. HW performed the literature review, synthesized results, and drafted the manuscript. DB conceived of the study and contributed to the manuscript. PD provided specialized content expertise, participated in the literature review, and contributed to the manuscript.

## Authors’ information

HWW holds a PhD in Policy Analysis from the Pardee RAND Graduate School, which supports an interdisciplinary approach to conducting decision analysis and solving complex problems. She also has a M.S. in Health Evaluation Sciences, with a focus on Health Services Research and Outcomes Evaluation. She has advised on HIT implementation efforts at a major academic medical center.

PKD is a Senior Principal Researcher at RAND and a Professor in the Pardee RAND Graduate School. He has published extensively on concepts, methods, and tools for supporting senior decision makers operating amidst great uncertainty. He has led development of ways to identify adaptive courses of action that can deal with both favorable or unfavorable developments.

DSB is a Research Scientist at RAND and an Associate Professor in the Department of Medicine at UCLA whose research focuses on the design and implementation of health information technologies. He is principal investigator of the Advancing Clinical Decision Support project. He is also co-director of the Biomedical Informatics Program of the UCLA Clinical and Translational Sciences Institute.

## Pre-publication history

The pre-publication history for this paper can be accessed here:

http://www.biomedcentral.com/1472-6947/12/90/prepub
